# Spermidine Attenuates Oxidative Stress-Induced Apoptosis via Blocking Ca^2+^ Overload in Retinal Pigment Epithelial Cells Independently of ROS

**DOI:** 10.3390/ijms22031361

**Published:** 2021-01-29

**Authors:** Da Hye Kim, Jeong-Hwan Kim, Hyun Hwangbo, So Young Kim, Seon Yeong Ji, Min Yeong Kim, Hee-Jae Cha, Cheol Park, Su Hyun Hong, Gi-Young Kim, Seh-Kwang Park, Ji-Won Jeong, Mi-Young Kim, Yung Hyun Choi, Hyesook Lee

**Affiliations:** 1 Anti-Aging Research Center, Dong-eui University, Busan 47340, Korea; 14983@deu.ac.kr (D.H.K.); hbhyun2003@naver.com (H.H.); 14731@deu.ac.kr (S.Y.K.); 14602@deu.ac.kr (S.Y.J.); ilytoo365@deu.ac.kr (M.Y.K.); hongsh@deu.ac.kr (S.H.H.); 2 Department of Biochemistry, Dong-eui University College of Korean Medicine, Busan 47227, Korea; 3 Research and Development Department, BGN CARE Co., Ltd., Busan 47195, Korea; genebio97@bgncare.com (J.-H.K.); psk11@bgncare.com (S.-K.P.); chief97@korea.ac.kr (M.-Y.K.); 4 Department of Parasitology and Genetics, Kosin University College of Medicine, Busan 49267, Korea; hcha@kosin.ac.kr; 5 Department of Molecular Biology, College of Natural Sciences, Dong-eui University, Busan 47340, Korea; parkch@deu.ac.kr; 6 Department of Marine Life Science, Jeju National University, Jeju 63243, Korea; immunkim@jejunu.ac.kr; 7 BGN Eye Clinic, Seoul 05551, Korea; 8 BGN Eye Clinic, Busan 47195, Korea; jjw22@bgncare.com

**Keywords:** cytosolic Ca^2+^, endoplasmic reticulum stress, oxidative stress, retinal pigment epithelial (RPE) cells, spermidine

## Abstract

Retinal pigment epithelial (RPE) cells occupy the outer layer of the retina and perform various biological functions. Oxidative damage to RPE cells is a major risk factor for retinal degeneration that ultimately leads to vision loss. In this study, we investigated the role of spermidine in a hydrogen peroxide (H_2_O_2_)-induced oxidative stress model using human RPE cells. Our findings showed that 300 μM H_2_O_2_ increased cytotoxicity, apoptosis, and cell cycle arrest in the G2/M phase, whereas these effects were markedly suppressed by 10 μM spermidine. Furthermore, spermidine significantly reduced H_2_O_2_-induced mitochondrial dysfunction including mitochondrial membrane potential and mitochondrial activity. Although spermidine displays antioxidant properties, the generation of intracellular reactive oxygen species (ROS) upon H_2_O_2_ insult was not regulated by spermidine. Spermidine did suppress the increase in cytosolic Ca^2+^ levels resulting from endoplasmic reticulum stress in H_2_O_2_-stimulated human RPE cells. Treatment with a cytosolic Ca^2+^ chelator markedly reversed H_2_O_2_-induced cellular dysfunction. Overall, spermidine protected against H_2_O_2_-induced cellular damage by blocking the increase of intracellular Ca^2+^ independently of ROS. These results suggest that spermidine protects RPE cells from oxidative stress, which could be a useful treatment for retinal diseases.

## 1. Introduction

Age-related macular degeneration (AMD), a multifaceted disease with demographic, environmental, and genetic risk factors, is among the most common causes of irreversible blindness in the world [1,2,3]. AMD progression occurs over an extended time period and its incidence rapidly increases in patients over 70 years old [1,4]. There are two major types of AMD: exudative or “wet” and non-exudative or “dry” [1,5]. Most patients with AMD have the “dry” form of the disease; the “dry” form is characterized by lipofuscin accumulation in the retinal pigment epithelial (RPE) cells and drusen formation beneath the RPE cells in Bruch’s membrane. In patients with “dry” AMD, these alterations of the normal retinal architecture lead to significant functional limitations but not loss of central vision [1,5]. In contrast, “wet” AMD comprises approximately 10–15% of all AMD cases and features choroidal neovascularization and abnormal blood vessel formation in macula. These abnormal neo-vessels eventually cause disciform scars, rupture, and blood leakage into the retina, and ultimately result in central vision loss [5]. Although the mechanism of AMD pathogenesis has yet to be fully understood, numerous studies suggest that chronic optic injury, choroidal vascular degeneration, and RPE aging are closely correlated to the progression of AMD [5,6]. In the pathogenesis of AMD, dysfunction or degeneration of the RPE cells occurs early in AMD progression and contributes to the formation of drusen deposits [6,7]. Accumulated evidence supports that protection of RPE cells from insult plays a critical role in the prevention of the pathological progression of AMD [6,8,9]. Therefore, loss of RPE cells can be predictive in the progression of AMD and protection from RPE injury could represent a potential strategy to delay the pathological progress of AMD.

The natural polyamines, including spermine, spermidine and putrescine, are involved in a host of biological processes and play a basic role in the regulation of growth and differentiation [10]. These polyamines are metabolites of ornithine and ubiquitous cellular components that have been reported to modulate the migration and proliferation of RPE cells [11,12,13]. Spermidine, specifically, is almost exclusively accumulated in glial cells within the central nervous system and retina [14,15]. Noro et al. demonstrated the neuroprotective effects of spermidine in a murine model of optic nerve injury, where results showed spermidine promoted optic nerve regeneration and the survival of retinal ganglion cells (RGC) [16]. Additionally, Noro’s group reported suppressed retinal degeneration and improved visual function via oral administration of spermidine in a murine glaucoma model [17]. Spermidine has also been reported to inhibit the action of reactive oxygen species (ROS), potentially acting as and endogenous ROS scavenger [18]. As relates to our chosen methodology, spermidine has been previously shown by Guo et al. [19] to suppress H_2_O_2_-induced RGC apoptosis; the same group also reported that the oral administration of spermidine reduced optic nerve demyelination and prevented RGC loss in a murine autoimmune encephalomyelitis model by acting as an antioxidant. Given previous findings, spermidine may prove effective in protecting both RPE cells and RGC from oxidative stress-mediated ocular diseases including glaucoma, traumatic optic neuropathy, and AMD. While previous studies clearly showed the potential therapeutic capacity of spermidine in the retina, the underlying mechanisms remain poorly explored, and further research into oxidative stress-mediated RPE degeneration is needed. To that end, we evaluated the effects of spermidine on oxidative stress-mediated RPE injury in hydrogen peroxide (H_2_O_2_)-stimulated human ARPE-19 cell line that is widely used as a cell model of the RPE [20], and identified the underlying mechanisms.

## 2. Results

### 2.1. Spermidine Attenuated H_2_O_2_-Induced Cytotoxicity in ARPE-19 Cells

**Figure 1:**
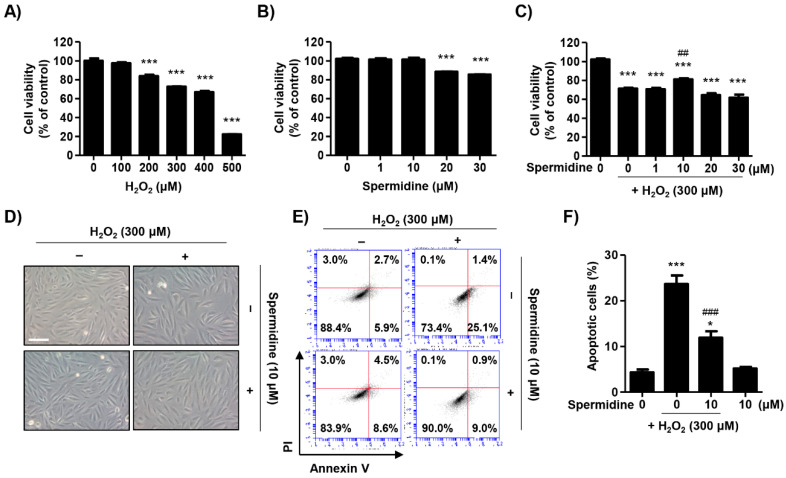
Effect of spermidine on apoptotic cell death in H_2_O_2_-stimulated ARPE-19 cells. (**A**,**B**) ARPE-19 cells were treated with various concentrations of H_2_O_2_ (**A**) or spermidine (**B**) for 24 h. (**C**–**F**) Cells were pre-treated with the indicated concentrations of spermidine for 1 h and then additionally incubated with 300 μM H_2_O_2_ for 24 h. (**A**–**C**) Cell viability was measured via 3-(4,5-dimethylthiazol-2-yl)-2,5-diphenyltetrazolium bromide (MTT) assay. Data are expressed as the mean ± SD (*n* = 5). *** *p* < 0.001 when compared to control. ## *p* < 0.01 when compared to H_2_O_2_-treated cells. (**D**) Morphological changes observed under an inverted microscope (scale bar; 75 μm). (**E**) Flow cytometry: annexin V and propidium iodine (PI). (**F**) The percentages of apoptotic cells were determined by counting the percentage of annexin V-positive cells. Data are expressed as the mean ± SD (*n* = 4). * *p* < 0.05 and *** *p* < 0.001 when compared to control. ### *p* < 0.001 when compared to H_2_O_2_-treated cells.

Oxidative stress was generated by the addition of various concentrations of H_2_O_2_ for 24 h and cell viability was measured via 3-(4,5-dimethylthiazol-2-yl)-2,5-diphenyltetrazolium bromide (MTT) assay. As shown in Figure 1A, H_2_O_2_ led to cytotoxicity at concentrations of 200 μM or more while cell viability dropped to approximately 74% at 300 μM. The IC_50_ value of H_2_O_2_ was 408.12 μM. Spermidine was also examined for cytotoxic effects and concentrations over 20 μM were found to be toxic (Figure 1B). To assess the protective effects of spermidine on H_2_O_2_-induced cytotoxicity, we treated cells with spermidine for 1 h prior to being treated with 300 μM H_2_O_2_ for 24 h. Figure 1C showed that pre-treatment with 10 μM spermidine significantly attenuated the decrease of cell viability induced by H_2_O_2_. However, spermidine does not have a protective effect against H_2_O_2_-induced cytotoxicity below 10 μM or over 20 μM. Morphologically, control ARPE-19 cells showed even density, spindle-shaped adherent monolayer growth, and stretched shapes (Figure 1D). In contrast, H_2_O_2_-treated cells were sparse in density with many cells detached, whereas pre-treatment with spermidine improved to the cell morphology such that it was comparable to controls. Next, we evaluated which mode of cell death was involved in H_2_O_2_-induced cytotoxicity and whether this event was regulated by spermidine. Results of flow cytometric analysis using annexin V/propidium iodine (PI) staining showed 300 μM H_2_O_2_ markedly enhanced the frequency of annexin V-positive cells to approximately 25%, but this increase was significantly suppressed by spermidine (Figure 1E,F). These results suggest that spermidine attenuates H_2_O_2_-mediated oxidative stress-induced apoptosis in ARPE-19 cells.

### 2.2. Spermidine Downregulated Extrinsic and Intrinsic Apoptosis Pathways in H_2_O_2_-Stimulated ARPE-19 Cells

**Figure 2:**
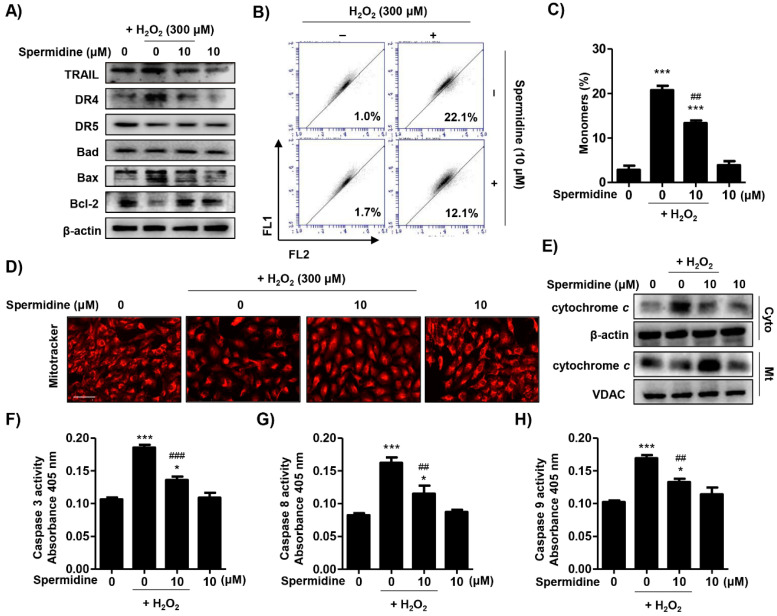
Effect of spermidine on extrinsic and intrinsic pathways in H_2_O_2_-induced apoptosis. Cells were pre-treated with or without 10 μM spermidine for 1 h and then additionally incubated with 300 μM H_2_O_2_ for 24 h. (**A**) The expression of extrinsic and intrinsic apoptosis-regulatory proteins was evaluated by Western blot analysis with whole cell lysates. Equal protein loading was confirmed by β-actin as an internal control. (**B**,**C**) Flow cytometry: 5,5′,6,6′-tetrachloro-1,1′,3,3′-tetraethyl-imidacarbocyanune iodide (JC-1). (**B**) Representative histograms. (**C**) The percentages of monomers were determined by counting the percentage of JC-1 green-positive cells. Data are expressed as the mean ± SD (*n* = 3). *** *p* < 0.001 when compared to control. ## *p* < 0.01 when compared to H_2_O_2_-treated cells. (**D**) Cells probed with 100 nM MitoTracker Red and observed under a fluorescence microscope. Scale bar: 200 μm. (**E**) Cytosolic and mitochondrial proteins were isolated and the expression of cytochrome c was detected by Western blot analysis. Cytochrome oxidase subunit VI (COX IV) and β-actin served as protein loading controls for the mitochondria and cytosol, respectively. The activities of caspase-3 (**F**), caspase-8 (**G**), and caspase-9 (**H**) were measured using caspase colorimetric assay kits. Data are expressed as the mean ± SD (*n* = 3). * *p* < 0.05 and *** *p* < 0.001 when compared to control. ## *p* < 0.01 and ### *p* < 0.001 when compared to H_2_O_2_-treated cells.

On the basis of spermidine’s suppression of H_2_O_2_-induced apoptosis in ARPE-19 cells, we investigated which apoptotic pathways were involved in this process. Figure 2A indicates that H_2_O_2_ upregulated the expression of death receptor 4 (DR4) and Bax, but downregulated the expression of anti-apoptotic Bcl-2. Spermidine reversed the altered expression of apoptosis regulator proteins following H_2_O_2_ exposure. To determine whether spermidine regulates the mitochondrial-mediated intrinsic apoptosis pathway, we evaluated mitochondrial functions, including mitochondrial membrane potential (MMP, ∆*Ψm*) and mitochondrial activity using 5,5′,6,6′-tetrachloro-1,1′,3,3′-tetraethyl-imidacarbocyanune iodide (JC-1) staining and MitoTracker Red staining, respectively. The outcome of flow cytometric analysis for JC-1 showed that H_2_O_2_ greatly promoted the frequency of JC-1 monomers, which indicates mitochondrial membrane potential (MMP, ∆*Ψm*) loss by mitochondrial membrane depolarization. As shown, pre-treatment with spermidine markedly suppressed H_2_O_2_-induced MMP (∆*Ψm*) loss (Figure 2B,C). Furthermore, Figure 2D indicates that the population of MitoTracker Red-positive cells, indicating healthy mitochondria, was decreased by H_2_O_2_, whereas it was recovered to control levels by spermidine. In addition, we investigated the effect of spermidine on changes in cytochrome c following MMP (∆*Ψm*) loss in H_2_O_2_-stimulated ARPE-19 cells. The immunoblotting results of Figure 2E show that expression of cytochrome c in H_2_O_2_-stimulated cells was increased in the cytoplasm as compared to the mitochondria; these results were reversed by pre-treatment with spermidine, indicating that spermidine has protective effects on cytochrome c release from the mitochondria induced by H_2_O_2_. Moreover, the activities of caspase-3, -8, and -9 were significantly increased by H_2_O_2_ stimulation, while substantially decreased by spermidine (Figure 2F–H). These results suggest that spermidine downregulated the extrinsic apoptosis pathway, including the increase in DR4 expression and caspase-8 activity upon H_2_O_2_ insult. Simultaneously, spermidine also acted on the intrinsic apoptosis pathway, displaying protective effects related to mitochondrial dysfunction-mediated MMP (∆*Ψm*) loss, Bcl-2 downregulation, cytochrome c release, and caspase-9 activation.

### 2.3. Spermidine Suppressed DNA Damage and Dysregulation of Cell Cycle Processes in H_2_O_2_-Stimulated ARPE-19 Cells

**Figure 3:**
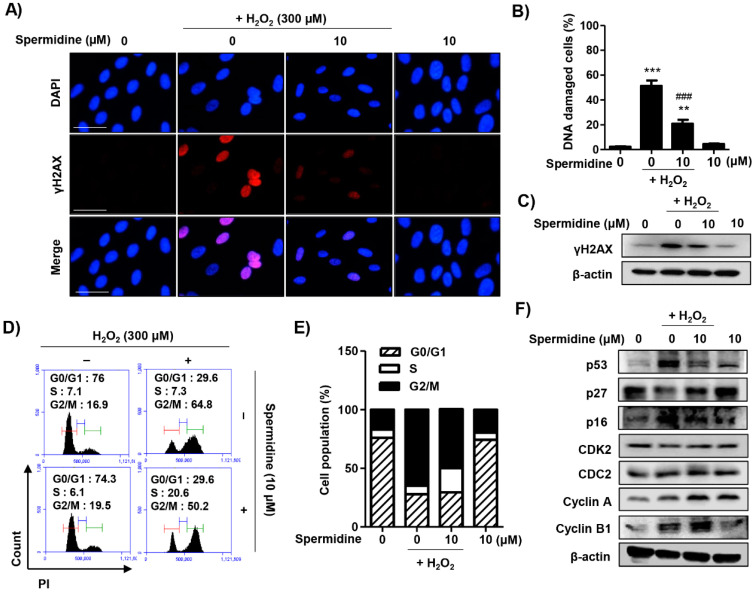
Effect of spermidine on DNA damage and cell cycle arrest in H_2_O_2_-stimulated ARPE-19 cells. Cells were pre-treated with or without 10 μM spermidine for 1 h and then additionally incubated with 300 μM H_2_O_2_ for 24 h. (**A**) The cells were immune-stained with γH2AX antibody (red) and visualized using a fluorescence microscope. 4′,6-Diamidino-2-phenylindole (DAPI) was used to counterstained the nuclei (blue). Scale bar: 75 μm. (**B**) The percentage of DNA-damaged cells in whole field. Data are expressed as the mean ± SD (*n* = 3). ** *p* < 0.01 and *** *p* < 0.001 when compared to control. ### *p* < 0.001 when compared to H_2_O_2_-treated cells. (**C**) Expression of γH2AX was determined by Western blot analysis. β-Actin was used as an internal control. (**D**,**E**) Flow cytometry: PI. (**D**) Representative histograms. (**E**) The average percentages of cells in each phase of the cell cycle are displayed (excluding sub-G1). (**F**) The expression of cell cycle-regulatory proteins was determined by Western blot analysis. β-Actin was used as an internal control.

To assess whether spermidine can decrease H_2_O_2_-induced DNA damage, we performed immunofluorescence analysis and immunoblots for γH2AX, a sensitive marker for DNA damage. As results indicate, spermidine greatly suppressed the increase in the expression of γH2AX following H_2_O_2_ insult (Figure 3A–C). We further examined the effect of spermidine on cell cycle progression in H_2_O_2_-stimulated ARPE-19 cells. The results of flow cytometric analysis for PI staining showed that H_2_O_2_ increased the distribution of cells in the G2/M phase, which was markedly decreased by spermidine treatment (Figure 3D,E). The result of Western blotting analysis for cell cycle regulators indicated that H_2_O_2_ upregulated the expression of p53, p16, cyclin A, and cyclin B1, while it downregulated the expression of p27; alterations in cyclin-dependent kinase 2 (CDK2) or cell division cycle gene 2 (CDC2, or CDK1) were not induced by H_2_O_2_ insult. Importantly, these changes to the expression of cell cycle regulators following H_2_O_2_ insult were noticeably restored by spermidine (Figure 3F). These results suggest that spermidine has a protective effect against H_2_O_2_-induced DNA damage and cell cycle arrest at the G2/M phase through the control of cell cycle regulators.

### 2.4. Spermidine Had Antioxidant Capacity But Did Not Regulate Intracellular ROS Generation in H_2_O_2_-Stimulated ARPE-19 Cells

**Figure 4:**
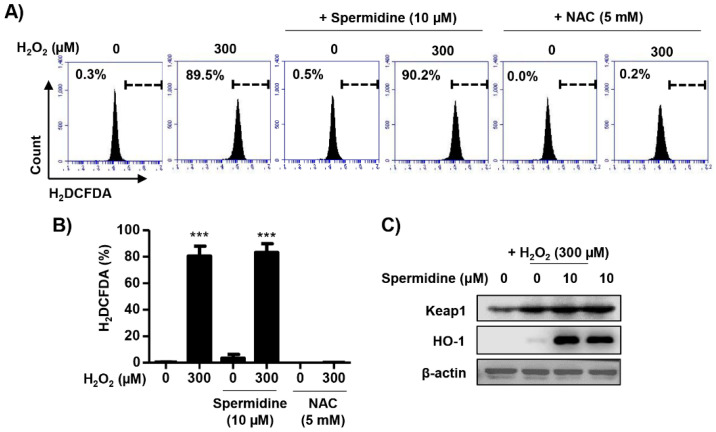
Effect of spermidine on intracellular reactive oxygen species (ROS) generation in H_2_O_2_-stimulated ARPE-19 cells. Cells were pre-treated with or without 10 μM spermidine or 5 mM N-acetyl-L-cysteine (NAC) for 1 h, and then treated 300 μM H_2_O_2_ for 30 min. Subsequently, cells were stained with 10 μM dichlorodihydrofluorescein diacetate (DCF-DA), and intracellular ROS generation was analyzed by flow cytometry. (**A**) Representative histograms. (**B**) DCF-DA fluorescence intensities were quantified. Data are expressed as the mean ± SD (*n* = 3). *** *p* < 0.001 when compared to control. (**C**) Expression of Kelch-like ECH-associated protein 1 (Keap1) and heme oxygenase-1 (HO-1) were evaluated by Western blot analysis with whole cell lysates. Equal protein loading was confirmed by β-actin as an internal control.

Next, to investigate whether the protective effects of spermidine on H_2_O_2_-induced apoptosis were due to the blocking of the oxidative stress, we observed the intracellular ROS levels using dichlorodihydrofluorescein diacetate (DCF-DA), a fluorescent-labeled probe. Results of flow cytometric analysis showed intracellular ROS production was significantly increased (≈90%) by H_2_O_2_ after 30 min and this increment was not altered by spermidine (Figure 4A,B). Pre-treatment with N-acetyl-L-cysteine (NAC), an ROS scavenger commonly used as a positive control, completely blocked intracellular ROS generation following H_2_O_2_. Meanwhile, H_2_O_2_ slightly upregulated the expression of Kelch-like ECH-associated protein 1 (Keap1) and heme oxygenase-1 (HO-1), which are enzymes with antioxidant properties induced by oxidative stress (Figure 4C). In spermidine-treated cells, the expression of Keap1 and HO-1 was markedly increased (Figure 4C). On the basis of these results, we determined that spermidine does not block H_2_O_2_-induced intracellular ROS generation despite its antioxidant capacity.

### 2.5. Spermidine Decreased Cytosolic Ca^2+^ Levels Released Due to Endoplasmic Reticulum (ER) Stress in H_2_O_2_-Stimulated ARPE-19 Cells

**Figure 5:**
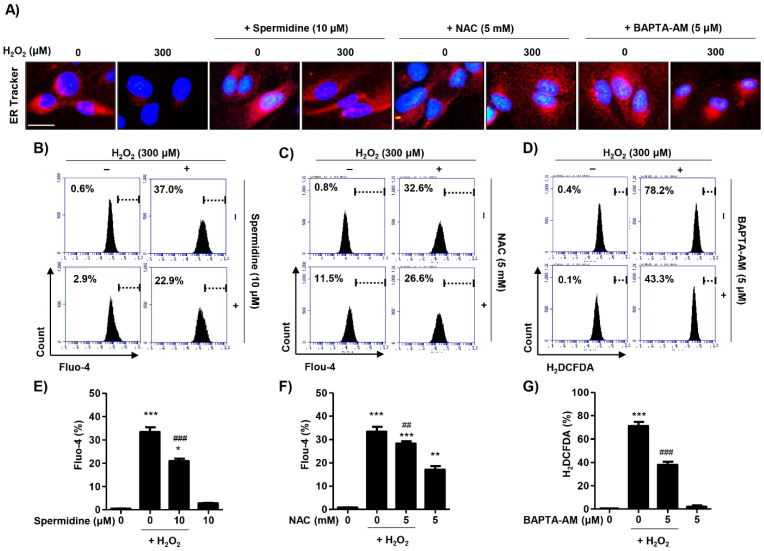
Effect of spermidine on cytosolic Ca^2+^ concentration in H_2_O_2_-stimulated ARPE-19 cells. Cells were pre-treated with or without 10 μM spermidine, 5 mM NAC, or 5 μM BAPTA-AM for 1 h and then additionally incubated with 300 μM H_2_O_2_ for 24 h. (**A**) Cells stained with 1 μM ER-Tracker Red dye and observed under a fluorescence microscope. DAPI was used to counterstained the nuclei (blue). Scale bar: 20 μm. (**B**,**C**,**E**,**F**) Cells were probed with 1 μM fluo-4 AM, an intracellular Ca^2+^ indicator, for 30 min, and then fluorescence intensity was monitored using a flow cytometer. (**B**,**C**) Representative histograms. (**E**,**F**) Quantified fluo-4 AM fluorescence intensities. Data are expressed as the mean ± SD (*n* = 3). * *p* < 0.05, ** *p* < 0.01, and *** *p* < 0.001 when compared to control. ## *p* < 0.01 and ### *p* < 0.001 when compared to H_2_O_2_-treated cells. (**D**,**G**) Cells were stained with 10 μM DCF-DA and intracellular ROS generation was analyzed using a flow cytometer. (**D**) Representative histograms. (**G**) Quantification of DCF-DA fluorescence intensity. Data are expressed as the mean ± SD (*n* = 3). *** *p* < 0.001 when compared to control. ### *p* < 0.001 when compared to H_2_O_2_-treated cells.

In order to determine whether spermidine is involved in the recovery of ER damage in H_2_O_2_-stimulated cells, we stained cultures with ER-Tracker Red. We observed the fluorescence expression of ER-Tracker Red was markedly suppressed in H_2_O_2_-treated cells as compared with control cells; spermidine substantially reversed these effects (Figure 5A). Next, we investigated the effect of spermidine on intracellular Ca^2+^ ([Ca^2+^]i) levels resulting from ER damage. ER stress frequently results in the release of Ca^2+^ from the interior of the ER, inducing cytosolic Ca^2+^ accumulation and triggering cell death [21]. Results of flow cytometry for N-[4-[6-[(Acetyloxy)methoxy]-2,7-difluoro-3-oxo-3H-xanthen-9-yl]-2-[2-[2-[bis[2-[(acetyloxy)methoxy]-2-oxoethyl]amino]-5-methylphenoxy]ethoxy]phenyl]-N-[2-[(acetyloxy)methoxy]-2-oxoethyl]glycine (acetyloxy)methyl ester (fluo-4 AM) staining demonstrated that spermidine significantly suppressed the increase in fluorescence intensity in fluo-4 AM-stained cells, indicating [Ca^2+^]i levels following H_2_O_2_ stimulation were decreased in the cytoplasm compared to H_2_O_2_ insult alone (Figure 5B,E). On the basis of the results showing spermidine suppressed [Ca^2+^]i levels, we sought to verify the relation between ROS and [Ca^2+^]i on H_2_O_2_-induced ER stress. To this end, we used 1,2 bis-(2-aminophenoxy) ethane-N, N, N’,N’-tetraacetic acid acetoxymethyl ester (BAPTA-AM), a membrane-permeable selective Ca^2+^ chelator, as well as NAC as positive controls. As shown in Figure 5A, NAC preserved the expression of ER-Tracker Red, indicating that the blocking of ROS repaired ER function. BAPTA-AM also improved the expression of ER-Tracker Red as compared to the H_2_O_2_-treated cells, but this expression was minor compared to spermidine or NAC (Figure 5A). As expected, the results of fluo-4 AM staining showed that NAC pre-treatment significantly suppressed fluorescence intensity compared with H_2_O_2_-treatment alone (Figure 5C,F). The [Ca^2+^]i levels were also elevated by NAC treatment in the absence H_2_O_2_ (Figure 5C,F). Additionally, intracellular ROS levels were markedly suppressed by BAPTA-AM treatment, suggesting that ROS and [Ca^2+^]i interacted by some mechanism (Figure 5D,G). These results suggest that H_2_O_2_ promotes intracellular ROS generation, resulting in increased [Ca^2+^]i levels due to ER stress. Interestingly, our results show that spermidine can act as a specific [Ca^2+^]i chelator in an ROS-independent manner.

### 2.6. Blocking of Cytosolic Ca^2+^ Levels Attenuated H_2_O_2_-Induced Cytotoxicity in ARPE-19 Cells

**Figure 6:**
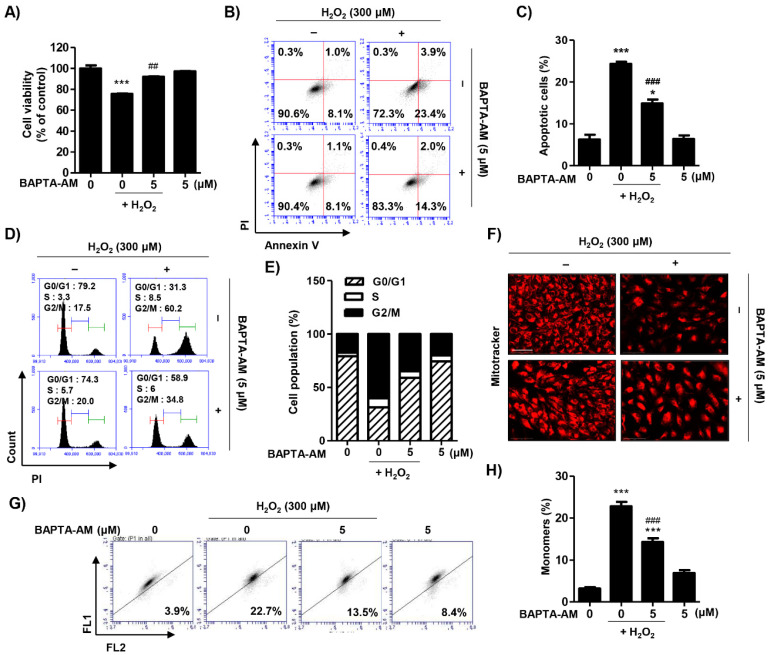
Effect of BAPTA-AM on H_2_O_2_-induced cellular damage in ARPE-19 cells. Cells were pre-treated with or without 5 μM BAPTA for 1 h and then additionally incubated with 300 μM H_2_O_2_ for 24 h. (**A**) Cell viability was measured via MTT assay. Data are expressed as the mean ± SD (*n* = 5). *** *p* < 0.001 when compared to control. ## *p* < 0.01 when compared to H_2_O_2_-treated cells. (**B**,**C**) Flow cytometry: annexin V and PI. (**B**) Representative histograms. (**C**) The percentages of apoptotic cells were determined by counting the percentage of annexin V-positive cells. Data are expressed as the mean ± SD (*n* = 4). * *p* < 0.05 and *** *p* < 0.001 when compared to control. ### *p* < 0.001 when compared to H_2_O_2_-treated cells. (**D**,**E**) Flow cytometry: PI. (**D**) Representative histograms. (**E**) The average percentages of cells in each phase of the cell cycle (excluding sub-G1). (**F**) Cells probed with 100 nM MitoTracker Red and observed under a fluorescence microscope. Scale bar: 200 μm. (**G**,**H**) Flow cytometry: JC-1. (**G**) Representative histograms. (**H**) The percentages of monomers were determined by counting the percentage of JC-1 green-positive cells. Data are expressed as the mean ± SD (*n* = 3). *** *p* < 0.001 when compared to control. ### *p* < 0.001 when compared to H_2_O_2_-treated cells.

To examine the role of spermidine as a specific intracellular Ca^2+^ chelator, we investigated the effect of BAPTA-AM on H_2_O_2_-induced cellular alteration. Pre-treatment with BAPTA-AM significantly improved cell viability and decreased apoptosis following H_2_O_2_ treatment (Figure 6A–C). Furthermore, BAPTA-AM markedly inhibited H_2_O_2_-induced cell cycle arrest at the G2/M phase (Figure 6D,E). Additionally, the mitochondrial function of BAPTA-AM-treated cells was also greatly improved compared to H_2_O_2_-stimualted cells (Figure 6F–H). These results suggest that ER stress-mediated intracellular Ca^2+^ increases play a critical role in H_2_O_2_-induced cytotoxicity.

## 3. Discussion

RPE cells are polarized epithelial cells that play a key role in retinal physiology including forming the outer blood–retinal barrier; transport of ions, water, nutrients, and metabolic end products; phagocytosis; and production of various growth factors [22]. Interestingly, the RPE is an ideal environment for the production of ROS [23]. The RPE contains an abundance of photosensitizers that generate reactive oxygen intermediates as a result of photochemical reactions and cellular metabolism [24]. Additionally, the process of phagocytosis by the RPE itself involves oxidative stress and leads to production of reactive oxygen intermediates [25]. Dysfunction and cell death in RPE cells are hallmarks of AMD; mechanistically, oxidative stress and reactive oxygen intermediates are believed to contribute to RPE cell death in AMD [26]. To identify the pathological mechanism of RPE dysfunction in AMD, numerous studies have evaluated RPE cell death in response to oxidative stress using pro-oxidants such as H_2_O_2_ and tert-butyl hydroperoxide (tBH) [27,28,29,30]. In the present study, we found that H_2_O_2_ led to cytotoxicity above 200 μM in ARPE-19 cells and accompanied apoptotic morphological changes. Furthermore, our findings indicate that H_2_O_2_-induced cytotoxicity caused apoptotic cell death in ARPE-19 cells. On the basis of these results, we established a model of oxidative stress-mediated RPE cell death using H_2_O_2_, and our findings demonstrated that spermidine significantly suppressed H_2_O_2_-induced RPE apoptosis (Figure 1).

Apoptosis can be executed by extrinsic or intrinsic pathways, which are mediated through cellular membrane death receptors and mitochondria, respectively [31]. The extrinsic pathway involves the formation of a death-inducing signaling complex and the recruitment of initiator caspases, which in turn activate the downstream effector caspases [32]. Meanwhile, the intrinsic pathway can be triggered by viral infection, UV, growth factor deprivation, inordinate ROS, or DNA damage, which lead to activation of proapoptotic Bcl-2 family proteins located in outer mitochondrial membrane [33]. Bax promotes the opening of the mitochondrial transition pores and subsequently induces the release of cytochrome c into the cytoplasm, which leads to the formation of an apoptosome and activation of effector caspases [33]. Both pathways activate the effector caspases, leading to the cleavage or degradation of cellular substrates including poly (ADP-ribose) polymerase and histones, ultimately leading to apoptotic cell death [31,32]. In the present study, we found that spermidine downregulated the activation of the extrinsic apoptosis pathway induced by H_2_O_2_, including DR4 expression and caspase-8 activity. Simultaneously, our findings demonstrated that spermidine also acts on intrinsic apoptosis pathway, including MMP (∆*Ψm*) loss, Bcl-2 down-expression, cytochrome c release to cytoplasm, and caspase-9 activation (Figure 2). Among the wide variety of factors that are instrumental in the etiology and pathogenesis of AMD, mitochondrial damage in RPE cells contributes significantly to RPE dysfunction [34]. In AMD, mitochondria are fragmented with a higher number of lesions, altered ATP synthase activity, as well as compromised protein expression and nuclear-encoded protein import [35,36]. Our findings demonstrate that oxidative stress induced by H_2_O_2_ caused mitochondrial damage and resulted in MMP (∆*Ψm*) loss as well as cytochrome c release; these conditions led to the activation of the intrinsic apoptosis pathway, which was suppressed by spermidine.

Cells are able to block the cell cycle transiently or irreversibly in response to stressful conditions. Apoptosis and cell cycle arrest commonly occur in response to DNA damage [37,38]. One response to DNA damage is the expression of γH2AX, which is an early sign of DNA damage induced by stalled replication [38]. The formation of γH2AX foci takes place immediately after the generation of a DNA break, as well as replication stalling or single-stranded DNA breaks [38]. When DNA is damaged, the G2 checkpoint inhibits cells from entering mitosis, thereby arresting the cell cycle at the G2/M phase, indicating that the damage of intracellular DNA is difficult to repair [39]. Progression of cell cycle is regulated by CDKs and their activity is coordinated by the binding of their essential regulatory subunits, cyclins [40]. The cyclin B1/CDC2 complex regulates cell cycle progression from the G2 to M phase, and cyclins accumulate steadily during the G2 phase while being rapidly eliminated as cells exit mitosis [41]. In this study, the suppression of γH2AX by spermidine demonstrated protective effects against H_2_O_2_-induced DNA damage in RPE cells. Furthermore, our findings provided data showing that H_2_O_2_ upregulated the expression of p53, p16, CDC2, cyclin A, and cyclin B1, whereas these changes to the expression of p53 and p16 following H_2_O_2_ were slightly downregulated by spermidine (Figure 3). In addition, our results suggest that spermidine has preventive effects against H_2_O_2_-induced cell cycle arrest at G2/M phase through partially control of cell cycle regulators (Figure 3). These results correspond with the findings of Liu et al. [42], reporting that oxidative damage by H_2_O_2_ triggered G2/M phase arrest of ARPE-19 cells through the regulation of cyclin B1. On the basis of our presented data, we can support the claim that spermidine suppresses oxidative stress-mediated DNA damage induced by H_2_O_2_, which in turn prevents cell cycle arrest in the G2/M phase.

As previous discussed, oxidative stress contributes to RPE cell death in AMD. In RPE cells, oxidative stress by ROS generation is mainly derived from the photo-oxidation of mitochondria, nicotinamide adenine dinucleotide phosphate (NADPH) oxidase, and the decline in ability to repair damaged organelles [23,43,44]. Several studies have suggested that H_2_O_2_ enhances the production of intracellular ROS in ARPE-19 cells [26,45,46], and our results agree with these reports (Figure 4). Of note, spermidine does not block H_2_O_2_-induced intracellular ROS generation despite its antioxidant actions. Although Rider et al. [18] demonstrated that spermidine inhibits the action of ROS and acts as an endogenous ROS scavenger, few studies have focused on the ROS scavenging effect of spermidine in RPE. In light of these reports, our findings are significant as we show that while spermidine has antioxidant capacity, it does not act as an ROS scavenger. On the basis of these results, we hypothesized that H_2_O_2_-induced oxidative stress is derived from not only intracellular ROS, but from other sources as well.

RPE cells are a target and source of various cytokines whose intracellular signaling cascades influence [Ca^2+^]i levels [22]. In addition, changes in [Ca^2+^]i are involved in normal RPE function, including transcellular fluid and ion transport, cell differentiation, and photoreceptor outer segment phagocytosis [22]. Homeostatic disorders in the calcium signaling system could represent a mechanism underlying apoptosis as changes in [Ca^2+^]i provide a chemical signal for early cell death [47,48]. As the ER stores [Ca^2+^]i for various physiological functions [49], experimental evidence suggests that ER dysfunction induced by oxidative stress in RPE cells is a key risk factor exacerbating the progression of AMD [50,51]. Yao et al. [52] demonstrated that H_2_O_2_-induced ER stress contributes to RPE cell apoptosis and that blocking ER stress inhibits H_2_O_2_-mediated RPE cell death. Moreover, Li et al. [53] suggested that ROS induced by H_2_O_2_ triggers RPE cell death through [Ca^2+^]i overload. In our research, H_2_O_2_ also increased [Ca^2+^]i through ER damage in ARPE-19 cells, but pretreatment with spermidine caused a significant decreased in cytoplasmic [Ca^2+^]i levels and ER damage. Furthermore, scavenging of ROS by NAC partially suppressed the increase in cytoplasmic [Ca^2+^]I, while blocking of [Ca^2+^]i by BAPTA-AM also decreased intracellular ROS levels (Figure 5). These findings suggest that ROS and [Ca^2+^]i interact during apoptotic processes in H_2_O_2_-stimulated ARPE-19 cells. Moreover, our results showed that blocking of [Ca^2+^]i suppressed H_2_O_2_-mediated apoptosis, cell cycle arrest, and mitochondrial damage (Figure 6). These data suggest that ER stress-mediated [Ca^2+^]i plays a critical role in H_2_O_2_-induced cytotoxicity and that spermidine acts as a [Ca^2+^]i chelator.

## 4. Materials and Methods

### 4.1. Chemicals and Reagents

Dichlorodihydrofluorescein diacetate (DCF-DA), 3-(4,5-dimethylthiazol-2-yl)-2,5-diphenyltetrazolium bromide (MTT), and fluo-4 AM were obtained from Invitrogen (Carlsbad, CA, USA). Annexin V fluorescein isothiocyanate (FITC)/propidium iodine (PI) double staining kit was purchased from BD Biosciences (San Diego, CA, USA). Caspase-3, -8, and -9 enzyme-linked immunosorbent assay (ELISA) kits were purchased from R&D Systems Inc. (Minneapolis, MN, USA). MitoTracker Red and ER-Tracker Red probe were obtained from Molecular Probes, Inc. (Eugene, OR, USA) and Thermo Fisher Scientific, Inc. (Rockford, IL, USA), respectively. BAPTA-AM, 4′,6-diamidino-2-phenylindole (DAPI), H_2_O_2_, 5,5′,6,6′-tetrachloro-1,1′,3,3′-tetraethyl-imidacarbocyanune iodide (JC-1), N-acetyl-L-cysteine (NAC), and spermidine (PubChem CID: 1102) were purchased from Sigma-Aldrich Chemical Co. (St. Louis, MO, USA). Bradford assay reagent and the mitochondrial fractionation kit were obtained from Bio-Rad Laboratories (Hercules, CA, USA) and Active Motif, Inc. (Carlsbad, CA, USA), respectively. Primary antibodies were purchased from Abcam, Inc. (Cambridge, MA, UK), Santa Cruz Biotechnology, Inc. (Santa Cruz, CA, USA), and Cell Signaling Technology (Danvers, MA, USA). Horseradish peroxidase (HRP)-conjugated secondary antibodies and Alexa Fluor 594-labeled donkey anti-rabbit immunoglobulin G (IgG) secondary antibody were obtained from Santa Cruz Biotechnology, Inc. and Invitrogen, respectively. All other reagents that were not specifically identified were purchased from Sigma-Aldrich Chemical Co.

### 4.2. Cell culture and Spermidine Treatment

ARPE-19 cells, a human RPE cell line, were purchased from the American Type Culture Collection (ATCC: Manassas, MD, USA), and the cells were maintained in Dulbecco’s modified Eagle’s medium: Nutrient Mixture F-12 (DMEM; WelGENE Inc., Daegu, Korea) supplemented with 10% fetal bovine serum (FBS), 100 U/mL penicillin, and streptomycin at 37 °C in a 5% CO_2_ incubator. Cells from passages 20–30 were used for all experiments. Spermidine was dissolved in dimethyl sulfoxide (DMSO) to 100 mM and diluted with culture medium to the final treatment concentrations before use in experiments.

### 4.3. Cell Viability Analysis

To measure the cytotoxicity of spermidine, we assessed cell viability via MTT assay. The cells were treated with various concentrations (0, 1, 10, 20, and 30 μM) of spermidine for 24 h. In order to assess the effect of spermidine, NAC, or BAPTA-AM upon oxidative stress, we pre-treated the cells with or without 10 μM spermidine, 5 mM NAC, or 5 μM BAPTA-AM for 1 h before being incubated for 24 h in the presence or absence of 300 μM H_2_O_2_. Afterwards, the cells were incubated with 0.5 mg/mL of MTT solution for 3 h before being dissolved in DMSO. Optical density was detected at 540 nm by a microplate reader (VERSA Max, Molecular Device Co., Sunnyvale, CA, USA) as previously described [54]. The cellular morphology was observed using an inverted microscope (Carl Zeiss, Oberkochen, Germany).

### 4.4. Flow Cytometric Analysis

Cells were pre-treated with or without 10 μM spermidine, 5 mM NAC, or 5 μM BAPTA-AM for 1 h before incubation for 24 h in the presence or absence of 300 μM H_2_O_2_. To measure apoptosis, we stained cells with FITC annexin V/PI for 20 min, according to the manufacturer’s protocol. The fluorescence intensity was detected using a flow cytometer (BD Biosciences), and FITC annexin V+/PI- cell populations were considered apoptotic [55]. In order to quantify the phase distribution of the cell cycle, we stained cells with 40 μg/mL PI for 30 min and analyzed them by flow cytometry [56]. To assess the MMP (∆*Ψm*), we loaded cells with 10 μM JC-1 for 20 min, and the frequency of JC-1 aggregates and monomers were analyzed [57]. For the intracellular calcium assay, cells were incubated with 1 μM fluo-4 AM probe for 30 min, and the fluorescence intensity was measured by flow cytometry [58].

### 4.5. Intracellular ROS Detection

Intracellular ROS production was assessed by DCF-DA staining as previously described [59]. In brief, cells were pre-treated with or without 10 μM spermidine, 5 mM NAC, or 5 μM BAPTA-AM for 1 h before incubation with 300 μM H_2_O_2_ for 30 min. Subsequently, 10 μM DCF-DA was added to the cell culture for 20 min and the stained images were acquired using a fluorescence microscope (Carl Zeiss).

### 4.6. Fluorescence Image Analysis

Cells were pre-treated with or without 10 μM spermidine, 5 mM NAC, or 5 μM BAPTA-AM for 1 h before incubation for 24 h in the presence or absence of 300 μM H_2_O_2_. To assess the function of mitochondria and the endoplasmic reticulum, we stained cells with 100 nM MitoTracker Red and 1 μM ER-Tracker Red probe, respectively, before observation using a fluorescence microscope (Carl Zeiss) per the manufacturer’s instructions.

### 4.7. Western Blot Analysis

ARPE-19 cells were pre-treated with or without 10 μM spermidine for l hour before incubation with 300 μM H_2_O_2_ for 24 h. Cells were harvested, lysed, and total proteins were analyzed using the Bradford protein assay kit (Bio-Rad Laboratories, Hercules, CA, USA). In a parallel experiment, mitochondrial and cytosolic proteins were extracted using a mitochondria isolation kit according to the manufacturer’s instructions. Equal amount of protein underwent sodium dodecyl sulfate polyacrylamide gel electrophoresis and were transferred to polyvinylidene difluoride membranes (Schleicher & Schuell, Keene, NH, USA). After blocking for 1 h, the membranes were incubated with specific primary antibodies at 4 °C overnight before incubation with the corresponding secondary antibodies for 1 h as previously described [60]. Information on the antibodies used is provided in Supplementary Table S1. The chemiluminescent bands were visualized by a Fusion FX Imaging System (Vilber Lourmat, Torcy, France).

### 4.8. Measurement of Caspases Activities

The cells were pre-treated with or without 10 μM spermidine for l hour before incubation with 300 μM H_2_O_2_ for 24 h and lysed. Caspase-3, -8, and -9 activities were determined using colorimetric assay kits according to the manufacturer’s instruction.

### 4.9. Immunofluorescence Analysis

Cells were transferred to a 4-well chamber slide (SPL Life Sciences Co., Pocheon, Korea) and incubated for 24 h, pre-treated with or without 10 μM spermidine for l hour, and then treated with 300 μM H_2_O_2_ for 24 h. The cells were then incubated with a γH2AX antibody (Cell Signaling Technology, Beverly, MA, USA, Cat No. 9718) at 4 °C overnight before being probed with an Alexa Fluor 594-labeled donkey anti-rabbit IgG antibody for 1 h in darkness. DAPI was used to counterstain the nuclei. The cells were mounted and observed using a fluorescence microscope (Carl Zeiss).

### 4.10. Statistical Analysis

All the experiments were performed by conducting each assay at least three times. The data were analyzed using GraphPad Prism 5.03 (GraphPad Software Inc., La Jolla, CA, USA) and are expressed as the means ± standard deviation (SD). The statistical analyses were conducted using analysis of variance (ANOVA) and Tukey’s post hoc test to examine between-group differences; *p* < 0.05 was considered significant.

## 5. Conclusions

**Figure 7:**
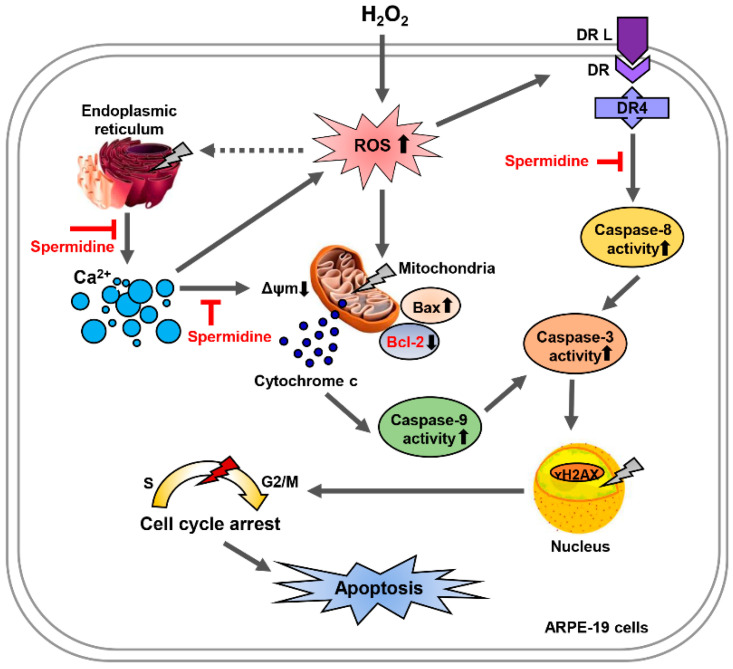
Spermidine attenuates H_2_O_2_-induced cellular dysfunction via suppression of Ca^2+^ signaling pathways in ARPE-19 cells. Oxidative stress by H_2_O_2_ triggers apoptosis through the intrinsic and extrinsic pathways in ARPE-19 cells. Spermidine markedly attenuated mitochondrial and nuclear dysfunction upon oxidative stress, inhibiting apoptosis. Spermidine suppresses intracellular Ca^2+^ levels and repairs ER damage, creating the anti-apoptotic effects. Nevertheless, while spermidine has antioxidant capacity, it does not down-regulate intracellular ROS generation during oxidative stress. Taken together, spermidine suppresses oxidative stress-induced cellular dysfunction via suppression of Ca^2+^ signaling pathways in ARPE-19 cells independently of ROS generation.

In conclusion, ARPE-19 cells treated with H_2_O_2_ underwent apoptosis through both the intrinsic and extrinsic pathways in response to cellular damage to the DNA, mitochondria, and ER. Pretreatment with spermidine caused a marked decrease in apoptosis and cell cycle arrest through the downregulation of ER stress-mediated [Ca^2+^]i overload, which is ROS-independent (Figure 7). However, further studies are needed to identify the role of spermidine on in the regulation of calcium channels in RPE. Our findings reveal that spermidine acts as a [Ca^2+^]i blocker in oxidative stress-induced RPE injury and offer a deeper understanding of spermidine’s clinical potential for the treatment of retinal disorders.

## Data Availability

The data presented in this study are available within the article and its supplementary material. Other data that support the findings of this study are available upon request from the corresponding authors.

## Supplementary Materials

The following are available online at https://www.mdpi.com/1422-0067/22/3/1361/s1: Table S1: Primary and secondary antibodies used for immunoblotting.
