# Supplementary material for: Spermidine Attenuates Oxidative Stress-Induced Apoptosis via Blocking Ca2+ Overload in Retinal Pigment Epithelial Cells Independently of ROS
Source: Int J Mol Sci. 2021 Jan 29;22(3):1361. doi: 10.3390/ijms22031361 (PMC7866386; doi:10.3390/ijms22031361)
Supplement: Supplementary file 1 [file ijms-22-01361-s001.pdf]

Supplementary Table S1. Primary and secondary antibodies used for immunoblotting.

Antibodies	Supplier	Item No.	Dilution
β-actin	Santa Cruz Biotechnology, Inc. (Santa Cruz, CA, USA)	sc-1615	1:2,000
Bad	Santa Cruz Biotechnology, Inc.	sc-8044	1:500
Bax	Santa Cruz Biotechnology, Inc.	sc-4780	1:500
Bcl-2	Santa Cruz Biotechnology, Inc.	sc-7382	1:500
Cdc2	Santa Cruz Biotechnology, Inc.	sc-54	1:1,000
Cdk2	Santa Cruz Biotechnology, Inc.	sc-6248	1:1,000
Cyclin A	Santa Cruz Biotechnology, Inc.	sc-239	1:1,000
Cyclin B1	Santa Cruz Biotechnology, Inc.	sc-245	1:1,000
Cytochrome C	Santa Cruz Biotechnology, Inc.	sc-7159	1:1,000
DR4	Santa Cruz Biotechnology, Inc.	sc-7863	1:1,000
DR5	Santa Cruz Biotechnology, Inc.	sc-166624	1:1,000
HO-1	Merck Millipore (Burlington, MA, USA)	374090	1:1,000
γH2AX	Cell Signaling Technology (Beverly, MA, USA)	#9718	1:1,000
Keap1	Bioss Antibodies (Woburn, MA, USA)	bs3648	1:1,000
p16	Santa Cruz Biotechnology, Inc.	sc-1661	1:1,000
p27	Cell Signaling Technology	#3686	1:1,000
p53	Cell Signaling Technology	#2524	1:1,000
TRAIL	Santa Cruz Biotechnology, Inc.	sc-8440	1:1,000
VDAC	Santa Cruz Biotechnology, Inc.	sc-390996	1:1,000
goat anti-mouse IgG-HRP	Santa Cruz Biotechnology, Inc.	sc-2005	1:1,500
goat anti-rabbit IgG-HRP	Santa Cruz Biotechnology, Inc.	sc-2004	1:1,500
